# *Elizabethkingia Meningoseptica:* An Emerging Nosocomial Pathogen Causing Septicemia in Critically Ill Patients

**DOI:** 10.5005/jp-journals-10071-23127

**Published:** 2019-02

**Authors:** Manoj Kumar Sahu, Uma Balasubramaniam, Bipin C, Sarvesh Pal Singh, Sachin Talwar

**Affiliations:** 1 Department of Cardiothoracic and Vascular Surgery, CN Centre, All India Institute of Medical Sciences, Ansari Nagar, New Delhi; 2-5 Department of Cardiac Anaesthesia, CN Centre, All India Institute of Medical Sciences, Ansari Nagar, New Delhi

**Keywords:** Elezabethkingia meningoseptica, Intensive care unit, Pediatric cardiac surgery, Sepsis

## Abstract

*Elizabethkingia meningoseptica* (*E. meningoseptica*), is an opportunistic Gram-negative bacteria, normally found in water and soil, notorious for causing nosocomial infections in extremes of ages and immunocompromised patients. It is now emerging as a serious nosocomial pathogen, intrinsically resistant to several commonly used antibiotics (e.g. beta-lactams, aminoglycosides, carbapenems and colistin etc. and a cause of high mortality in critically ill patients in intensive care units (ICUs).

We report the first case of *E. meningoseptica* sepsis in a 5 month old child after open heart surgery for transposition of great arteries, initially on extracorporeal membrane oxygenation, then prolonged mechanical ventilation, with various invasive devices, inotropes and exposed to broad spectrum antibiotics in our ICU. The case highlights the potential risk factors responsible for E.meningoseptica sepsis. Its unusual pattern of resistance to many commonly used antibiotics makes this organism difficult to treat.

**How to cite this article:**

Sahu MK, Balasubramaniam U *et al*. *Elizabethkingia Meningoseptica:* An Emerging Nosocomial Pathogen Causing Septicemia in Critically Ill Patients. Indian J of Crit Care Med 2019;23(2):104-105.

## INTRODUCTION

*Elizabethkingia meningoseptica* (*E. meningoseptica*) is a non-motile, non-fermentative, oxidase positive, Gram-negative Bacillus described by Elizabeth O. King in 1959 ^1^. It is not known to reside in human hosts, but notorious for causing nosocomial infections (NI) including meningitis, pneumonia and bacteremia in extremes of ages and immunocompromised hosts^2^.

The organism colonizes sink basins, taps, and become a potential reservoir for NIs in the hospital environment^3^. Colonization of the contaminated medical devices involving fluids, ventilators, breathing circuits, endotracheal tubes, humidifiers, intravascular devices, etc. have been documented as of serious concern^4–6^. This organism is resistant to many commonly used antibiotics (beta-lactams, carbapenems, aminoglycosides, etc.), posing treatment challenges^7^. Antimicrobial susceptibility data on this pathogen remains limited, with no established breakpoints by the Clinical and Laboratory Standards Institute criteria for minimum inhibitory concentration interpretation^8^. We report a case of *E. meningoseptica* septicemia in a pediatric cardiac surgical patient with multiple risk factors eventually leading to poor outcome.

## CASE REPORT

A five month female child weighing five kilos with transposition of great arteries, intact ventricular septum with regressed left ventricle underwent elective primary arterial switch operation. She was shifted to the cardiac surgical intensive care unit (CSICU) with open sternum on extracorporeal membrane oxygenation (ECMO) support because of severe left ventricular dysfunction with left ventricular ejection fraction(LVEF) of ~10%, failing to wean off cardiopulmonary bypass (CPB). The child was on intravenous (iv) inotrope (adrenaline 0.05 μg/kg/min, dobutamine 5mcg/kg/min) and vasodilator (nitroglycerine 0.5 μg/kg/min and phenoxybenzamine 0.5 μg/kg/8 hrly) infusions.

The infant was started on combined antibiotics with tazobactum+piperacilin 50mg/kg/iv/6hrly, amikacin15 μg/kg/iv/24 hrly and vancomycin 10 mg/kg/iv/8 hrly. The hemodynamics, urine output were maintained over time as her left ventricular function improved (LVEF 30%). ECMO was weaned off and sternum was closed after 72 hours. Vancomycin was stopped after sternal closure. Echo assessment revealed persisting LV dysfunction and mild mitral regurgitation. The child remained intubated, ventilated and inotropic support was continued alongwith the vasodilators. Enteral feeding was started with mother's milk through nasogastric tube. All the invasive devices like chest drainage tubes, central venous catheter, urinary catheter and pacing wires were maintained with sterile precautions. Her hemogram, and serum biochemistry remained unremarkable till 8th postoperative day (POD). She continued to be on low cardiac outputstate requiring inotropes and could not be weaned off ventilator. She was tracheostomized on POD 9. On POD 10, she developed fever, raised total leucocyte counts (13,700/mm^3^) with neutrophil predominance(78%), elevated procalcitonin levels (69.19 ng/mL), low platelets (56000/mm ^3^). Her systolic blood pressure (SBP) and urine output decreased to <40 mm Hg and <0.5 mL/kg, respectively and she required noradrenaline @ 0.1 μg/kg/min. Peritoneal dialysis (PD) was initiated. Blood, urine and endotracheal aspirate samples were sent for culture. *Klebsiella pneumoniae* was isolated from endotracheal aspirate and treated with colistin 30000 units/kg/8 hrly and imipenem 20 mg/kg/8 hrly from POD 12. However, the child did not improve appreciably. Repeat culture was done on POD 14 isolated *E. meningoseptica*, which showed resistance to most of the antibiotics including colistin. This was the first occasion we experienced this rare pathogen in our CSICU. We immediately warranted strict asepsis and followed institutional protocol for infection control to curtail spread of infection. Treatment was started with ciprofloxacin 50 mg/ iv/12 hrly and trimethoprim + sulfamethoxazole 15 mg/iv/8hrly. The child showed signs of improvement for a while but worsened again on POD 20 with deteriorated hemodynamics, requiringhigh dose vasopressor support, increased blood lactate and metabolic acidosis, progressed to septic shock with multiorgan failure and succumbed.

## DISCUSSION

*E. menigoseptica* is a rare Gram-negative opportunistic pathogen, known to cause nosocomial infection in susceptible patients with risk factors like prolonged ventilation, multiple invasive devices, PD, ECMO, exposed to multiple higher antibiotics, infected with other multidrug resistant gram negative pathogens and long ICU stay, etc. The organism is known for late onset of infection, 5-50 days post hospitalization^9^. It is resistant to a broad range of regularly used antimicrobials including polymixins. However, it is susceptible to antibiotics used to treat gram-positive bacteria i.e. rifampicin, ciprofloxacin, vancomycin and trimethoprim-sulfamethoxazole ^9^. Vancomycin alone or in combination with rifampicin, has in the past been successful in treating *E. meningoseptica*^10^, but its efficacy has been questioned by recent studies ^11^.

In our case, a post cardiotomy infant on prolonged mechanical ventilation developed sepsis. The time from hospital admission to isolation of organism was 16 days. *Klebsiella pneumoniae* was isolated from respiratory secretions initially and was treated with colistin and imipenem. While the treatment for *klebsiella* was being followed up with cultures of tracheal secretions, the second sample from respiratory secretions grew *E. meningoseptica* which was resistant to almost all routine antibiotics including colistin. Sepsis with *klebsiella* and treatment with colistin both might have acted as selective factors along with the other risk factors like younger age, delayed sternal closure, ECMO, PD catheter, urinary catheter, prolonged mechanical ventilation, invasive lines and chest drains. All these allowed *E. meningoseptica* to emerge in the environmental vicinity of the patient and cause nosocomial pneumonia in this already immuncompromised infant.

A recent case series by Govindaswamy *et al*. reported 12 days (10-25 days) of median time from admission to isolation of *E. meningoseptica*. All their patients had a history of recent hospitalization, at the time of sepsis, they were on mechanical ventilation in ICU. All cases received 2 or more antibiotics which included colistin and carbapenems. All isolates were resistant to commonly used antibiotics. Mortality rate was high (75%) in their study^12^. Our patient had findings similar to the above study.

*E. meningoseptica* has unusual resistance patterns and mechanisms. The reappearance of septic shock was probably attributed to this organism. The child was already infected with *Klebsiella*, probably it increased the susceptibility of this child to *E. meningoseptica* sepsis and unusually the child never responded to the treatment and succumbed to septic shock.

In view of its multidrug-resistant nature, its propensity to infect infants, as in our case, and to spread in the hospital environment, its prompt diagnosis in clinical samples and sensitivity testing along with reinforcement of standard infection control measures are essential to reduce outbreaks.

## CONCLUSION

The clinicians working in the ICUs need to be aware of this kind of rare organism as a potential pathogen especially in infants undergoing surgery for complex congenital heart disease with a protracted postoperative course. Also risk factors like ECMO, longer mechanical ventilation, PD, invasive lines and sepsis caused by multidrug resistant Gram-negative bacteria, exposure to broad spectrum antibiotics, etc. should warrant prompt initiation of therapy along with strict reinforcement of standard infection control measures.
